# Cell Signaling Pathways in Mammary Carcinoma Induced in Rats with Low versus High Inherent Aerobic Capacity

**DOI:** 10.3390/ijms20061506

**Published:** 2019-03-26

**Authors:** Tymofiy Lutsiv, John N. McGinley, Elizabeth S. Neil, Henry J. Thompson

**Affiliations:** Cancer Prevention Laboratory, Colorado State University, Fort Collins, CO 80523, USA; tymofiy.lutsiv@colostate.edu (T.L.); john.mcginley@colostate.edu (J.N.M.); elizabeth.neil@colostate.edu (E.S.N.)

**Keywords:** cell signaling, mammary cancer, inherent aerobic capacity

## Abstract

An inverse association exists between physical activity and breast cancer incidence and outcomes. An objective indicator of an individual’s recent physical activity exposure is aerobic capacity. We took advantage of the fact that there is an inherited as well as inducible component of aerobic capacity to show that experimentally induced mammary cancer is inversely related to inherent aerobic capacity (IAC). The objective of this study was to determine whether cell signaling pathways involved in the development of mammary cancer differed in rats with low inherent aerobic capacity (LIAC, *n* = 55) versus high inherent aerobic capacity (HIAC, *n* = 57). Cancer burden was 0.21 ± 0.16 g/rat in HIAC versus 1.14 ± 0.45 in LIAC, *p* < 0.001. Based on protein expression, cancer in LIAC animals was associated with upregulated glucose utilization, and protein and fatty acid synthesis. Signaling in cancers from HIAC rats was associated with energy sensing, fatty acid oxidation and cell cycle arrest. These findings support the thesis that pro-glycolytic, metabolic inflexibility in LIAC favors not only insulin resistance and obesity but also tumor development and growth. This provides an unappreciated framework for understanding how obesity and low aerobic fitness, hallmarks of physical inactivity, are associated with higher cancer risk and poorer prognosis.

## 1. Introduction

Lifestyle choices represent risk factors for breast cancer, which is the second leading cause of death in the United States among women [1]. Alcohol consumption, unhealthy diet, sedentary life style, and obesity have been associated with breast cancer morbidity and mortality [2]. In contrast, regular physical activity is considered a beneficial behavior that is associated not only with reduced breast cancer risk, but also decreased susceptibility to the development of obesity, cardiovascular disease and type 2 diabetes, which are common comorbidities in breast cancer patients [3,4,5]. The range of physical activity in human populations is quite diverse making it of interest to determine what accounts for protective activity [6].

Assessment of physical activity levels has an inherent bias due to human error in self-reporting the amount, type and intensity of physical activity [7]. However, an individual’s aerobic capacity—which can be measured as maximal oxygen uptake—is a more objective and reliable factor that predicts exercise-induced health benefits [8]. Low levels of aerobic capacity have been found to be a strong predictor of all-cause morbidity and mortality. Although higher levels of aerobic capacity are associated with better cancer prognosis, the evidence is limited [9], and thus, requires more thorough investigation in order to identify the conditions of positive response to a particular program of exercise.

Regular moderate to high intensity physical activity leads to increased maximal oxygen uptake levels and corresponds to the induced component of aerobic capacity. However, improvement in aerobic fitness has a heritable component which accounts for about 50% of the variability among individuals in their responsiveness to the same defined exercise training program [10,11]. The impact of this heritable component of aerobic capacity on disease outcomes, particularly cancer, is poorly understood.

In order to provide a means for the systematic investigation of intrinsic fitness, a model was developed using artificial two-way selection in the outbred N:NIH strain of rat [12]. Rats were selected for their performance in a graded treadmill running test. This effort resulted in two rat strains, high inherent aerobic capacity (HIAC) and low inherent aerobic capacity (LIAC). Not only did these animals diverge in their intrinsic aerobic capacity and ability to improve aerobic fitness in response to exercise training, they also exhibited different susceptibility to disease risks that span from metabolic to cardiovascular and neurodegenerative diseases [13]. LIAC animals develop insulin resistance and are prone to obesity due to altered mitochondrial function, energy utilization and energy expenditure. These metabolic disorders appear to emerge from lower metabolic flexibility associated with LIAC rats [14].

Such disparity in maintaining metabolic homeostasis by HIAC/LIAC strains indicates their suitability for studying molecular mechanisms that underlie the nature of inherent aerobic capacity (IAC) and its connection to health and disease [13]. However, the majority of work on aerobic capacity has been done on muscle tissue as it is the predominant hub linking physical activity to its effects on cells [15,16,17,18,19]. Nevertheless, low IAC has been reported to result in pathophysiologic changes in other tissues [20,21,22]. Recently, our laboratory showed that low IAC was associated with higher risk of experimentally induced mammary carcinogenesis in comparison to rats with high inherent aerobic capacity [23].

HIAC and LIAC animals respond differently to the carcinogen, 1-methyl-1-nitrosurea (MNU) administration—LIAC animals are more prone to the formation of breast cancer unlike their HIAC counterparts. Different rates of carcinogenic response in these two strains were observed in the absence of any physical training, which is consistent with an intrinsic origin of these effects [23]. Our laboratory demonstrated different levels of protein expression within the mammary gland of animals with low or high IAC: HIAC animals featured more activated AMPK and downregulated AKT pathways compared with LIAC, and resulted in decreased mTOR signaling pathway activity [23]. The signaling activity in the mammary gland that distinguished between LIAC and HIAC is characteristic of the differences in metabolic flexibility associated with type 2 diabetes and obesity [14]. The purpose of this investigation was to determine the characteristics of the mammary cancers that occurred in the experiment previously reported [23] and investigate whether the pattern of signaling activity observed in mammary carcinoma in HIAC and LIAC is similar to or distinct from that observed in the mammary gland.

## 2. Results

### 2.1. Characteristics of Induced Tumors

**Figure 1:**
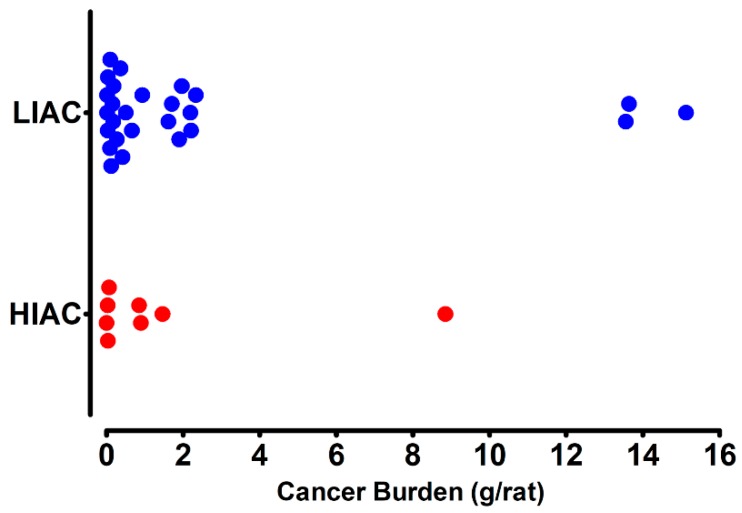
Effect of inherent aerobic capacity on tumor burden. Total tumor mass per rat was computed and is displayed for rats with high (HIAC, red dot) or low (LIAC, blue dot) inherent aerobic running capacity. Cancer burden was 0.21 ± 0.16 g/rat, *p* < 0.001, in HIAC, which was significantly lower than in LIAC (1.14 ± 0.45, *p* < 0.001).

Tumor size and hormone receptor status are indicators of breast cancer prognosis. As shown in Figure 1, cancer burden (tumor mass per animal) was markedly higher in LIAC rats: 1.14 ± 0.45 g/rat in LIAC vs. 0.21 ± 0.16 g/rat in HIAC, *p* < 0.001.

**Figure 2:**
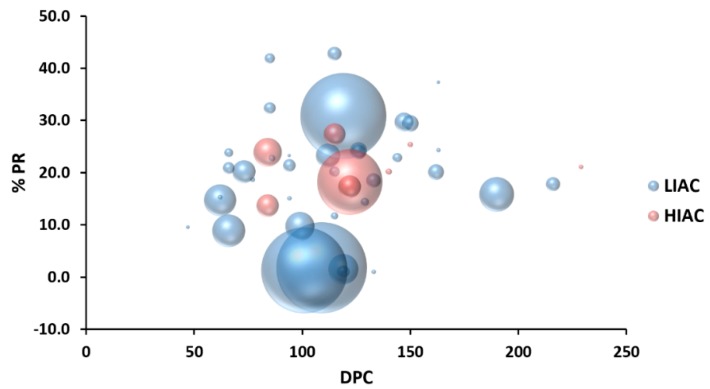
Effect of inherent aerobic capacity on tumor hormone receptor status. For all palpable mammary carcinomas, percent of cells staining positive for progesterone receptor (% PR) was determined. This data, plus the mass of the tumor determined gravimetrically at necropsy and the days post carcinogen (DPC) that the mass was initially detected by palpation were used to construct a bubble chart with the size of each bubble reflecting relative tumor mass.

Tumors histologically classified as adenocarcinomas were immunohistochemically stained for presence of ER/PR (estrogen and progesterone receptors, respectively) and PR staining was considered an indicator of functional hormone receptor signaling (HRS). The percent of PR positive cells was graphed as a bubble chart with tumor mass and the day post carcinogen on which the carcinoma was detected (Figure 2). This graph summarizes the impact of IAC on cancer incidence (14.0% vs. 47.3% *p* < 0.001) and multiplicity (0.18 vs. 0.85 cancers/rat, *p* < 0.0001), HIAC versus LIAC, respectively, as previously reported, as well as showing the mean percent progesterone receptor staining cells. HRS did not vary statistically by aerobic capacity status, although visual inspection of the graph shows wide variation in staining in the LIAC group indicating the presence of both steroid hormone receptor responsive and non-responsive tumors in the LIAC group. The HRS of HIAC tumors was more uniform and indicative of tumors with a more favorable prognosis. These findings suggest differences in the cells of origin within the mammary epithelium that give rise to mammary carcinomas and point to the potential value of studying how aerobic capacity affects cell differentiation within the mammary gland.

### 2.2. Mammary Tumor Protein Expression

While the number of mammary tumors suitable for analysis in the HIAC group was limited because of tumor size, three HIAC tumors were available and matched to three tumors in the LIAC group based on weight, time of detection post carcinogen injection, and HRS status. Our underlying assumption was that protein expression patterns would be similar in LIAC versus HIAC since the same chemical was used to induce mammary cancer in both groups.

**Figure 3:**
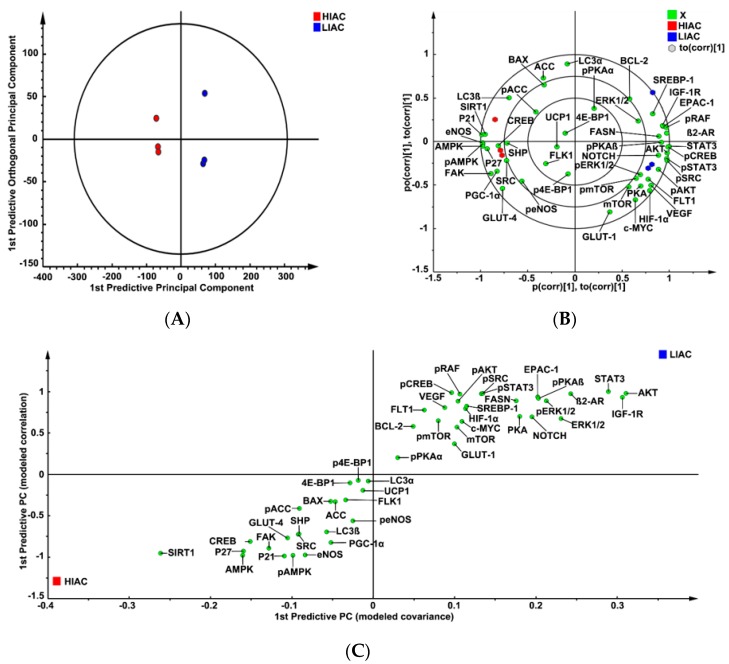

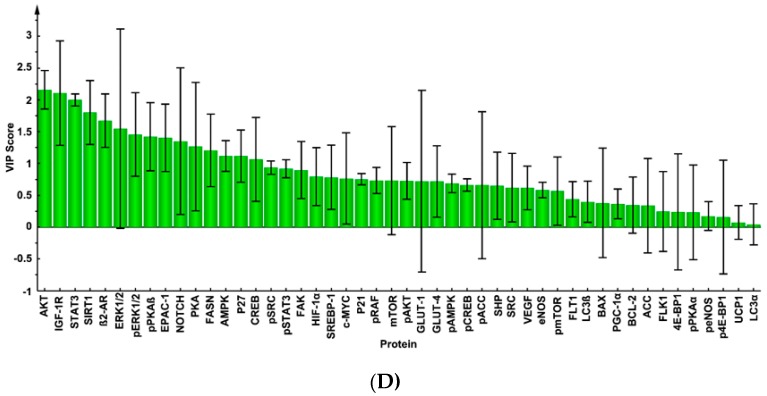
Analysis of protein expression in the mammary carcinoma using a supervised clustering algorithm. Effects of high (HIAC) or low (LIAC) inherent aerobic running capacity on patterns of protein expression in the mammary carcinoma (*n* = 3/group) were assessed by multivariate discriminant analysis. Initially, inherent clustering patterns were determined by unsupervised analysis through principle component analysis (PCA) and complete separation of treatment groups was observed. (**A**) To determine contributing sources of variation, the scatter plot represents supervised analysis of the 2-class Orthogonal Projections to Latent Structures for Discriminant Analysis (OPLS-DA) model, which rotates the model plane to maximize separation due to class assignment. Complete separation of the two classes was observed. The circle on the graph is the 95% confidence ellipse for the OPLS-DA analysis; it was computed by the SIMCA software that was used for the analysis based on the Hotelling T^2^ statistic discriminant model. Samples lying outside the 95% confidence interval are considered outliers; (**B**) to determine the proteins responsible for class separation multivariate analysis was used to construct a biplot that identified influential proteins responsible for the separation between classes. The circles on the plot are graphed by SIMCA to assist with the visualization of the location of scores and loadings at the 0.5, 0.75 and 1.0 coordinates on the X and Y axes; (**C**) an S-plot was constructed by plotting modeled correlation in the first predictive principal component against modeled correlation from the first predictive component. Upper right and lower left regions of S-plots contain candidate proteins with both high reliability and high magnitude discriminatory proteins; (**D**) to determine the statistical reliability of the effects, variable importance plots were generated in which jack-knifed confidence intervals (JKCI) were created on the magnitude of covariance in the first component for the analytes assessed. Proteins with JKCI including 0 were considered not to account for separation.

The protein expression data were mean-centered, Pareto-scaled, and evaluated using unsupervised principle component analysis (PCA). There was complete separation between groups with 100% classification accuracy, thus favoring the dissimilarity hypothesis. To determine contributing sources of variation, a supervised analysis of the 2-class Orthogonal Projections to Latent Structures for Discriminant Analysis (OPLS-DA) model was performed (Figure 3A). This analysis rotates the model plane to maximize separation due to class assignment. Complete separation of the two classes was observed. The model had two components with cross-validated predictive ability, Q2(Y) = 46.7%, and total explained variance, R2(X) = 81.8%. The same data set was then subjected to supervised OPLS-DA and cross-validated predictive ability of the model, Q2(Y), increased to 99.9% with 74% of total variance, R2(X), explained. The biplot for this model (Figure 3B) shows not only the scores plot depicting the separation among tumors in LIAC versus HIAC, but also the proteins most influential in accounting for the observed separation. These effects were further quantified via S-plot analysis (Figure 3C) and variable importance for projection analysis (VIP; Figure 3D).

The results of this analysis indicated that CKIs, p21 and p27 were significantly higher in HIAC tumors, whereas higher expression of pERK1/2, pAKT, pmTOR was associated with LIAC tumors. Relative to apoptosis, higher BAX expression was associated with HIAC tumors while higher BCL-2 expression was associated with LIAC tumors, although those associations, i.e., as indicated by the VIP plot, were weak relative to the effects exerted by p21 or p27. The focus of analysis shifted to examining what regulatory proteins were associated with the tumor locations on the scores plot using the vector locations on the biplot. Examination revealed higher levels of activated AMPK and SIRT1 in immediate proximity to the score values of tumors and vectors for p21 and p27, whereas patterns of protein expression characteristic of Notch and activated PKA co-localized with LIAC tumors and the factors associated with glycolytic phenotype. Higher levels of proteins associated with de novo lipid synthesis (FASN, SREBP1) were also associated with LIAC.

### 2.3. Mammary Gland Protein Expression

**Figure 4:**
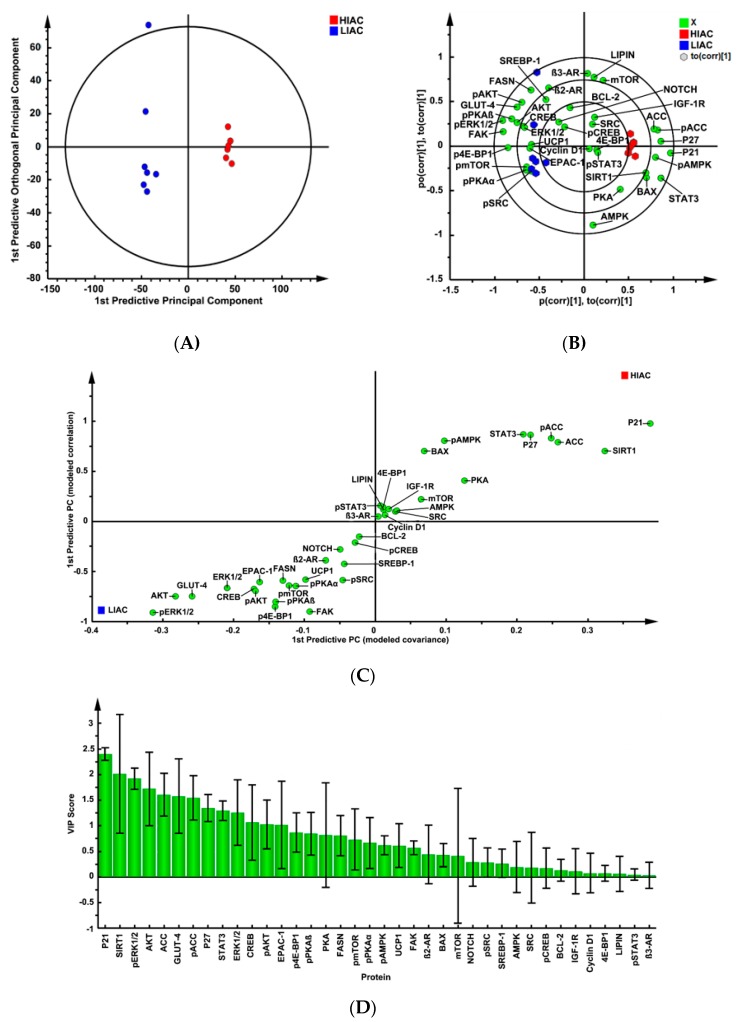
Analysis of protein expression in the mammary gland using a supervised clustering algorithm. Effects of high (HIAC) or low (LIAC) inherent aerobic running on patterns of protein expression in the mammary gland (*n* = 7/group) were assessed by multivariate discriminant analysis. Initially, inherent clustering patterns were determined by unsupervised analysis through PCA and complete separation of treatment groups was observed. (**A**) To determine contributing sources of variation, the scatter plot represents supervised analysis of the 2-class OPLS-DA model, which rotates the model plane to maximize separation due to class assignment. Complete separation of the two classes was observed. The circle on the graph is the 95% confidence ellipse for the OPLS-DA analysis; it was computed by the SIMCA software that was used for the analysis based on the Hotelling T^2^ statistic discriminant model. Samples lying outside the 95% confidence interval are considered outliers; (**B**) to determine the proteins responsible for class separation multivariate analysis was used to construct a biplot that identified influential proteins responsible for the separation between classes. The circles on the plot are graphed by SIMCA to assist with the visualization of the location of scores and loadings at the 0.5, 0.75 and 1.0 coordinates on the X and Y axes; (**C**) an S-plot was constructed by plotting modeled correlation in the first predictive principal component against modeled correlation from the first predictive component. Upper right and lower left regions of S-plots contain candidate proteins with both high reliability and high magnitude discriminatory proteins; (**D**) to determine the statistical reliability of the effects, variable importance plots were generated in which JKCI were created on the magnitude of covariance in the first component for the analytes assessed. Proteins with JKCI including 0 were considered not to account for separation.

Mammary gland was analyzed for expression of proteins similar to those evaluated in mammary tumors. Analyses were confined to pathology-free mammary tissue which is comprised predominately of mammary epithelial cells, adipocytes and the stroma in which the mammary epithelium is embedded. The expression data were mean-centered and Pareto-scaled. Data were subjected to unsupervised PCA and complete separation was observed with 100% classification accuracy supporting the dissimilarity hypothesis. The same data set was then subjected to supervised OPLS-DA and cross-validated predictive ability of the model Q2(Y) was 96.9% with 65.3% of total variance, R2(X), explained (Figure 4A).

The biplot for this model (Figure 4B), S-plot analysis (Figure 4C), and VIP analysis (Figure 4D) indicated that p21, p27, BAX, activated AMPK, ACC, and SIRT1 were more highly expressed in HIAC versus LIAC and localized with HIAC mammary glands in the biplot. On the other hand, proteins associated with mTOR activation (pmTOR, pAKT, p4E-BP1) and activation of PKA (β2-AR, pPKAα/β, pSRC) localized with LIAC mammary glands and where they were more highly expressed. However, some of the proteins involved in PKA-mediated signaling (pSTAT3, pCREB) localized in the center of the biplot indicating that they had little effect in accounting for separation, which is also apparent in the S-plot and the VIP plot.

## 3. Discussion

Given that clinical evidence indicates an inverse association between breast cancer risk and intensity/volume of physical activity as well as that both exercise intensity and/or volume are positively associated with aerobic capacity, our mechanistic studies focused on the pathways that have been implicated in linking induced aerobic capacity to the development of chemically induced mammary carcinogenesis in the rat [23,24,25,26,27,28,29]. The fact that differences in protein expression in mammary tumors and mammary glands were sufficient to permit 100% discrimination between LIAC and HIAC indicates that paracrine and autocrine regulation are likely to be playing a role in accounting for differences in the carcinogenic response [23].

The strongest evidence for differential expression between LIAC and HIAC was for oncogenic signaling, i.e., AKT, mTOR, ERK1/2, signatures of G1/S arrest, such as p21 and p27, as well as lipid metabolism (ACC, FASN, SREBP1) based on the VIP rankings (Figure 3D). Consistent with these observations, activation of energy sensors, such as AMPK and SIRT1, was detected in HIAC carcinomas, both of which have been implicated in the resistance of HIAC in other disease model systems [30,31]. On the other hand, the activation of EPAC- and PKA-related signaling was apparent in mammary carcinomas of LIAC.

Our analyses show that mammary glands of animals with LIAC exhibit an anabolic signature with stronger correlation to pathways that are central in oncogenic transformation, such as cAMP-PKA/EPAC-1, AKT-mTOR, ERK1/2, SREBP1, FASN. Their expression was even higher in tumors of these animals. Protein expression profiles characteristic of cell proliferation, cell survival, and accumulation of lipids and proteins were predominant in LIAC. Overall, the expression of AKT, SIRT1, β2-AR, p21, p27, PKA, ERK1/2, EPAC-1 were the most influential factors in tumors based on classification by IAC. The magnitude of expression of these proteins was greater in tumors than mammary gland, a finding consistent the pathological differences that exist between these two tissues [32,33]. Generally, HIAC had a phenotype associated with higher rates of energy expenditure in mammary tumors as well as mammary gland, a finding consistent with improved metabolic flexibility. Accordingly, we postulate that IAC is associated with the main factors of cellular level regulation of metabolic flexibility, such as AMPK and SIRT1; AKT, mTOR, ERK1/2, PKA, Notch signaling pathways, and their targets that regulate the switch between fatty acid and glucose metabolism [34]. Our results are consistent with genomic studies in skeletal muscles that identified AMPK and mTOR signaling networks as well as energy sensing, cell cycle, hypoxia and other elements as candidate mechanisms associated with IAC [11].

**Figure 5:**
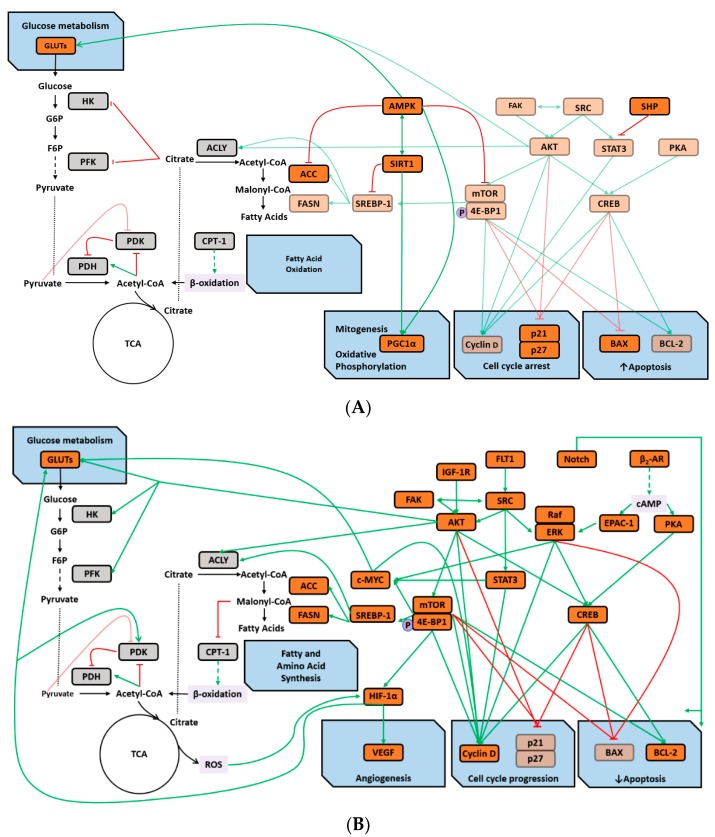
Metabolic and signaling pathway crosstalk in tissues of animals with high inherent aerobic capacity (HIAC) and animals with low inherent aerobic capacity (LIAC). (**A**) Greater fatty acid oxidation is upregulated by activity of AMPK and SIRT1 at the expense of glucose metabolism. Acetyl-CoA from TCA and β-oxidation activate PDK and impedes glycolysis from glucose oxidation by inhibiting PDH, whereas post TCA citrate inhibits HK and PFK attenuating glycolysis and undergoes ACLY-mediated conversion into acetyl-CoA. Further carboxylation of cytosolic acetyl-CoA into malonyl CoA is impeded by AMPK-induced inhibition of ACC. AMPK also downregulates lipogenesis (FASN, ACC) by inhibiting mTOR-SREBP-1 pathway and induces glucose uptake by upregulating GLUTs. SREBP-1 is also downregulated by SIRT1 which is in crosstalk with AMPK. In addition, inactive SRC, STAT3, AKT and PKA-CREB factors and corresponding signaling pathways are associated with HIAC tissues. Altogether, such metabolic and signaling pattern leads to cell cycle arrest (via p21 and p27), induction of apoptosis (via increase in BAX and decrease in BCL-2 levels), as well as increase in mitochondrial function (via PGC 1α); (**B**) glucose metabolism is upregulated in LIAC tissues at the expense of fatty acid oxidation leading to production of ROS and anabolic metabolism: AKT signaling induces glycolysis via GLUTs, HK, PFK; GLUTs are also induced by AKT-mTOR-mediated upregulation of HIF-1α and c-MYC factors; AKT-mTOR-SREBP1-stimulated ACLY and ACC promotes conversion of malonyl-CoA which inhibits CPT-1 and subsequent β oxidation of fatty acids. In turn, AKT-mTOR-SREBP1-stimulated FASN utilizes malonyl-CoA and increases lipogenesis. AKT is downstream target of SRC and FAK which can affect activity of one another. SRC also activates STAT3 and ERK1/2. cAMP, produced from stimulation of β2-AR stimulation, activates EPAC-1, which also activates ERK1/2. cAMP also activates PKA. Common downstream target of AKT, ERK1/2, and PKA is CREB. All of these as well as Notch signaling pathway induce angiogenesis (via VEGF), cell cycle progression (via stimulation of cyclin D and inhibition of p21 and p27) and downregulate apoptosis (by inducing BCL-2 and lowering BAX). Factors detected in our experimental analyses are depicted in orange, whereas those obtained from literature analyses are in gray.

Collectively our findings are consistent with the thesis that the maintenance of a pro-glycolytic environment in which glucose is the preferred fuel source is a metabolic state favored in tumors, in multiple tissues of obese, insulin resistant organisms, and in animals and people with low aerobic capacity. As such, this provides an unappreciated framework for better understanding how obesity and low aerobic fitness, hallmarks of physical inactivity, are associated with higher cancer risk and poorer cancer prognosis. The molecular underpinnings of this hypothesis, based on integrated analysis of the literature and our experimental findings, are summarized in Figure 5.

## 4. Materials and Methods

### 4.1. Animals

Eight breeding pairs of rats categorized by phenotype as having HIAC or LIAC from generation 29 of selection were mated using a Poiley rotation breeding scheme. Female offspring of these breeding pairs were used for the carcinogenesis experiment as previously reported [35]. Briefly, 21-day old female LIAC and HIAC rats were injected intraperitoneally with 1-methyl-1-nitrosurea (MNU, 70 mg/kg, Ash Stevens, Detroit, MI, USA) according to published procedure [36]. A total of 55 LIAC and 57 HIAC female rats were assigned to each group serially as they became available from the breeding colony. Rats were group-housed (three per cage) in solid bottomed polycarbonate cages and fed a standard laboratory diet for rodents (Harlan 2918 Teklad Lab Animal Diet, Madison, WI). All rats remained sedentary, i.e., physical activity was limited to spontaneous activity within their group housed setting throughout the study. Animal rooms were maintained at 22 ± 1 °C with 50% relative humidity and a 12-h light/12-h dark cycle. Rats were weighed weekly and palpated for the detection of mammary tumors twice per week starting from three weeks post carcinogen. The study was terminated 33 weeks post carcinogen injection.

At necropsy, rats were euthanized, skinned and the pelt was examined under translucent light for detectable mammary pathologies at 5× magnification. All detectable mammary gland pathologies were excised, weighed, and a cross section prepared for histological classification according to published criteria [37]. The remainder of each mammary pathology weighing more than 100 mg was snap frozen in liquid nitrogen. This experimental protocol number 11-2611A was reviewed and approved by the Institutional Animal Care and Use Committee on 22/04/2011 and conducted according to the committee guidelines at Colorado State University.

### 4.2. Immunohistochemistry

Immunohistochemical assessment of hormone receptor status, ER/PR receptor status of mammary carcinomas, was determined using our previously published procedure [38]. Our laboratory has evaluated a number of ER antibodies in the past and the best performing ER antibody currently available for rat tissue yielded significantly weaker staining intensity compared to the more robust signal intensity achieved with PR staining; quantification of PR is more reproducible because the signal to noise ratio is high. A polymer-based detection method, Envision+ (Agilent Technologies/Dako, Carpinteria, CA, USA) was used amplify the signal for both ER and PR antibodies, which is more sensitive than conventional detection, e.g., streptavidin-biotin complex. Since it is known that signaling through the estrogen receptor is required for progesterone receptor expression, quantification was limited to the progesterone receptor. Primary antibodies used for ER/PR staining are listed in Supplementary Table S1.

### 4.3. Protein Expression

Rather than conducting non targeted profiling for identification of candidate mechanisms to explain the differential cancer susceptibility of LCR and HCR, a comprehensive list of signaling molecules and pathways linking energetics, particularly physical activity and the development of breast cancer in the rat mammary cancer model, was compiled based on our published work [23,24,25,26,27,28,29]. This list provided the rationale for subsequent experiments on tissue. Data from our Western blots from other studies (references [23,24,25,26,27,28,29]) indicated an *n* = 7 has > than 80% power to detect effects sizes of the magnitude reported in those references. Mammary gland was selected, using a randomized approach, from animals in which no mammary pathologies were detectable at 5× magnification when the gland was excised, spread out on transparency film and trans illuminated at necropsy before it was flash frozen in liquid nitrogen. Each tumor was taken from a different animal. It was decided to use a balanced design in which each HIAC tumor was matched with an LIAC tumor with a similar time of occurrence, mass, and PR staining.

Mammary tumor and mammary gland tissue lysate were prepared and Western blotted as previously reported [29]. Briefly, tissue was homogenized in lysis buffer [40 mM Tris-HCl (pH 7.5), 1% Triton X-100, 0.25 M sucrose, 3 mM EGTA, 3 mM EDTA, 50 μM β-mercaptoethanol, 1 mM phenyl-methylsulfonyl fluoride, and complete protease inhibitor cocktail (Calbiochem, San Diego, CA, USA)]. The lysates were centrifuged at 7500× *g* for 10 min at 4 °C and supernatant fractions collected and stored at –80 °C. Supernatant protein concentrations were determined by the Bio-Rad protein assay (Bio-Rad, Hercules, CA, USA). Western blotting was performed as described previously. Briefly, 40 µg of protein lysate per sample was subjected to 8%–16% sodium dodecyl sulfate-polyacrylamide gradient gel electrophoresis (SDS-PAGE) after being denatured by boiling with SDS sample buffer [63 mM Tris-HCl (pH 6.8), 2% SDS, 10% glycerol, 50 mM DTT, and 0.01% bromophenol blue] for 5 min. After electrophoresis, proteins were transferred to a nitrocellulose membrane. The amounts of target proteins were determined using specific primary antibodies (Supplementary Table S2), followed by treatment with the appropriate peroxidase-conjugated secondary antibodies and visualized by LumiGLO reagent Western blotting detection system. The chemiluminescence signal was captured using a ChemiDoc densitometer (Bio-Rad). All Western blot signals were within the linear range as indicated by Chemidoc software and protein expression was actin-normalized (Supplementary Table S3).

### 4.4. Data Evaluation

Differences in cancer immunohistochemical staining for the steroid hormone receptors and for cancer burden were evaluated by the nonparametric Kruskal Wallis test [39]. Data from Western blot analyses were evaluated via unsupervised and supervised multivariate techniques for hypothesis generation per our previously published approach [35]. PCA was used to determine relationships between LIAC and HIAC with no prior knowledge of class membership. OPLS-DA, a supervised, class-based method where class membership is assigned and used to elicit maximum data separation, was applied using the class information to partition variation into predictive and orthogonal components. The contribution of each component partitioned into between-class (predictive) and within-class (orthogonal) variance was also estimated [40,41,42,43]. A scatter plot of the first two score vectors for the PCA models were drawn, along with 95% confidence ellipses based on Hotelling’s multivariate T2, to identify outliers that might bias the results. For OPLS-DA, class separation was shown in several ways:

Biplot was used to co-chart scores and loadings from the OPLS-DA model. Observations (proteins) situated near variables (HIAC/LIAC) are high in those variables. Variables close to the plot origin are poorly described by the model components. S-plot was constructed to identify influential proteins in the separation of treatment groups with heavily influential features separating from other features at the upper right and lower left tails of the model space [41,43]. Variable importance in the projections (VIP) plot expresses the influence of class assignment, i.e., LIAC versus HIAC, on every variable. The VIP for each variable expressed with its respective 95% confidence interval, can be compared with one another. VIP greater than 1 are considered most relevant to accounting for class separation. All multivariate analyses were done using SIMCA-P + v.12.0.1 (Umetrics, Umea, Sweden).

## 5. Conclusions

The lower cancer risk and better prognosis associated with being of normal weight and aerobically fit are linked to metabolic flexibility in which the oxidation of glucose and fatty acids fluctuate with the energy status of the organism. On the other hand, increased cancer risk and poorer cancer prognosis that are linked to obesity and low aerobic fitness are associated with preferential oxidation of glucose irrespective of the energy status of the organism. Our experiments indicate the merit of: (1) determination of the dominant cellular process involved in inhibiting carcinogenesis with primacy given to examining effects of LIAC/HIAC status on the G1/S checkpoint in the cell cycle; (2) examination of the role of mTOR network activity, a signaling cascade deregulated in the majority of human breast, with a focus on regulation of mTOR via sensors of both intracellular and extracellular energy/nutrient status; (3) investigation of the role of cAMP-EPAC-1/PKA network activity, which has received limited attention in the breast cancer field, but that plays a central role in energy homeostasis, particularly aspects that relate to fuel availability to support skeletal muscle contraction during physical activity.

## Supplementary Materials

Supplementary materials can be found at https://www.mdpi.com/1422-0067/20/6/1506/s1.

## Abbreviations

4E-BP1	4E Binding Protein 1
ACC	Acetyl-CoA-carboxylase
ACLY	ATP-citrate Lyase
AKT	Protein Kinase B
AMPK	AMP-activated kinase
BAX	BCL-2-associated X protein.
β2-AR	β2-adrenergic Receptor
BCL-2	B cell lymphoma/leukaemia-2 protein
cAMP	Cyclic Adenosine Monophosphate
CKI	Cyclin-dependent Kinase Inhibitor
c-MYC	Oncogenic transcription factor
CPT-I	Carnitine Palmitoyltransferase I
CREB	Cyclic-AMP-responsive element binding protein
DPC	Days Post Carcinogen
EPAC	Guanine-nucleotide exchange proteins activated by cAMP 1
ER	Estrogen Receptor
ERK1/2	Mitogen-activated protein kinase, extracellular signal regulated kinases 1 and 2
FAK	Focal Adhesion Kinase
FASN	Fatty Acid Synthase
FLT1	Vascular endothelial growth factor receptor 1
GLUTs	Glucose transporters
HIAC	High Inherent Aerobic Capacity
HIF-1α	Hypoxia Inducible Factor-1α
HK	Hexokinase
HRS	Hormone Receptor Signaling
IAC	Inherent Aerobic Capacity
IGF-1R	Insulin Growth Factor 1 Receptor
JKCI	Jack-knifed Confidence Intervals
LIAC	Low Inherent Aerobic Capacity
MNU	1-methyl-1-nitrosurea
mTOR	Mammalian Target of Rapamycin
OPLS-DA	Orthogonal Projections to Latent Structures for Discriminant Analysis
p21	Cyclin-dependent kinase inhibitor 1
p27	Cyclin dependent kinase inhibitor 1B
p4E-BP1	Phospho 4E Binding Protein
pAKT	Phospho protein Kinase B
PCA	Principle Component Analysis
pCREB	Phospho cyclic-AMP-responsive Element Binding protein
PDH	Pyruvate Dehydrogenase
PDK	Pyruvate Dehydrogenase Kinase
pERK1/2	Phosopho mitogen-activated protein kinase, extracellular signal regulated kinases 1 and 2
PFK	Phosphofructokinase
PGC	Peroxisome proliferator-activated receptor gamma coactivator
PGC-1α	Peroxisome proliferator-activated receptor gamma coactivator 1-alpha
PKA	Protein kinsae A
pmTOR	Phospho mammalian target of rapamycin
pPKAα/β	Protein kinsae A alpha / beta
PR	Progesterone Receptor
pSRC	Phospho non-receptor tyrosine kinase SRC
pSTAT3	Phospho signal transducer and activator of transcription 3
RAF	Rapidly Accelerated Fibrosarcoma kinase
ROS	Reactive Oxygen Species
SIRT-1	Silent mating type information regulation 2 homolog 1
SRC	Non-receptor tyrosine kinase SRC
SREBP1	Sterol Regulatory Element-binding Protein 1
STAT3	Signal and transducer of transcription 3
TCA	Tricarboxylic Acid
VEGF	Vascular Endothelial Growth Factor;
VIP	Variable Importance Projection
