# Supplementary material for: Cell Signaling Pathways in Mammary Carcinoma Induced in Rats with Low versus High Inherent Aerobic Capacity
Source: Int J Mol Sci. 2019 Mar 26;20(6):1506. doi: 10.3390/ijms20061506 (PMC6470785; doi:10.3390/ijms20061506)
Supplement: Supplementary file 1 [file ijms-20-01506-s001.zip › Supplementary_Tables_S1_S2.docx]

**Supplementary Table S1.** Primary Antibodies Used in Immunohistochemistry

| **Antibody** | **Vendor** | **Catalog No.** |
| --- | --- | --- |
| ER (1D5) | Agilent Tech. / Dako | M7047 |
| PR (PR88) | Biogenex | MU328-UC |

**Supplementary Table S2.** Primary Antibodies Used in Western Blots

| **Antibody** | **Vendor** | **Catalog No.** |
| --- | --- | --- |
| p4EBP1 (Thr37/46) | Cell Signaling | 9459 |
| 4E-BP1 | Cell Signaling | 9452 |
| pACC (Ser79) | Cell Signaling | 3661 |
| ACC | Cell Signaling | 3676 |
| pAkt (Ser473) | Cell Signaling | 4058 |
| Akt | Cell Signaling | 9272 |
| pAMPK (Thr172) | Cell Signaling | 2535 |
| AMPK | Cell Signaling | 2603 |
| β2-AR | Santa Cruz Biotechnology | SC-9042 |
| β3-AR | Abcam | AB101095 |
| BAX | Cell Signaling | 2772 |
| BCL2 | Cell Signaling | 2870 |
| c-MYC | Cell Signaling | 9402 |
| pCREB (Ser133) | Cell Signaling | 9198 |
| CREB | Cell Signaling | 4820 |
| Cyclin D1 | Abcam | AB134175 |
| pENOS (Ser1177) | Cell Signaling | 9571 |
| eNOS | Cell Signaling | 9572 |
| EPAC-1 | Cell Signaling | 4155 |
| pERK1/2 (Thr202/Tyr204) | Cell Signaling | 9101 |
| ERK1/2 | Cell Signaling | 9102 |
| FAK | Cell Signaling | 3285 |
| FASN | Cell Signaling | 3180 |
| FLK1 | Santa Cruz Biotechnology | SC-315 |
| FLT1 | Santa Cruz Biotechnology | SC-316 |
| GLUT-1 | Santa Cruz Biotechnology | SC-7903 |
| GLUT-4 | Santa Cruz Biotechnology | SC-7938 |
| HIF-1α | Novus | NB-100-479 |
| IGF1R-α | Santa Cruz Biotechnology | SC-712 |
| LC3α/ß | Cell Signaling | 4108 |
| LC3β | Cell Signaling | 2775 |
| LIPIN-1 | Santa Cruz Biotechnology | SC-50049 |
| pmTOR (Ser2448) | Cell Signaling | 2971 |
| mTOR | Cell Signaling | 2972 |
| Notch1 | Cell Signaling | 4380 |
| P21 | Santa Cruz Biotechnology | SC-397 |
| P27 | Abcam | 32034 |
| PGC-1α | Cell Signaling | 2178 |
| PKA | Santa Cruz Biotechnology | SC-365615 |
| pPKAα/β (Thr198) | Santa Cruz Biotechnology | SC-32968 |
| pc-RAF (Ser338) | Cell Signaling | 9427 |
| SHP | Santa Cruz Biotechnology | SC-15283 |
| SIRT-1 | Cell Signaling | 9475 |
| pSRC (Ser17) | Cell Signaling | 12432 |
| SRC | Cell Signaling | 2123 |
| SREBP-1 | Santa Cruz Biotechnology | SC-365513 |
| pSTAT3 (Tyr705) | Cell Signaling | 9145 |
| STAT3 | Cell Signaling | 9139 |
| UCP1 | Abcam | AB23841 |
| VEGF | Santa Cruz Biotechnology | SC-152 |
